# Outcome after PSMA-PET/CT-based salvage radiotherapy for nodal recurrence after radical prostatectomy

**DOI:** 10.1007/s00259-021-05557-z

**Published:** 2021-10-10

**Authors:** Paul Rogowski, Christian Trapp, Rieke von Bestenbostel, Chukwuka Eze, Ute Ganswindt, Minglun Li, Marcus Unterrainer, Mathias J. Zacherl, Harun Ilhan, Leonie Beyer, Alexander Kretschmer, Peter Bartenstein, Christian Stief, Claus Belka, Nina-Sophie Schmidt-Hegemann

**Affiliations:** 1 Department of Radiation Oncology, University Hospital, LMU Munich, Marchioninistr. 15, 81377 Munich, Germany; 2 Department of Radiation Oncology, University Hospital, Medical University Innsbruck, Innsbruck, Austria; 3 Department of Radiology, University Hospital, LMU Munich, Munich, Germany; 4 Department of Nuclear Medicine, University Hospital, LMU Munich, Munich, Germany; 5 Department of Urology, University Hospital, LMU Munich, Munich, Germany; 6 German Cancer Consortium (DKTK), Munich, Germany

**Keywords:** Prostate cancer, Nodal recurrence, PSMA PET/CT, Salvage radiotherapy, Nodal radiotherapy, ENRT

## Abstract

**Purpose:**

Nodal recurrent prostate cancer (PCa) represents a common state of disease, amenable to local therapy. PSMA-PET/CT detects PCa recurrence at low PSA levels. The aim of this study was to evaluate the outcome of PSMA-PET/CT-based salvage radiotherapy (sRT) for lymph node (LN) recurrence.

**Methods:**

A total of 100 consecutive patients treated with PSMA-PET/CT-based salvage elective nodal radiotherapy (sENRT) for LN recurrence were retrospectively examined. Patients underwent PSMA-PET/CT scan due to biochemical persistence (bcP, 76%) or biochemical recurrence (bcR, 24%) after radical prostatectomy (RP). Biochemical recurrence-free survival (BRFS) defined as PSA < post-RT nadir + 0.2 ng/ml and distant metastasis-free survival (DMFS) were calculated using the Kaplan–Meier method and uni- and multivariate analysis was performed.

**Results:**

Median follow-up was 37 months. Median PSA at PSMA-PET/CT was 1.7 ng/ml (range 0.1–40.1) in patients with bcP and 1.4 ng/ml (range 0.3–5.1) in patients with bcR. PSMA-PET/CT detected 1, 2, and 3 or more LN metastases in 35%, 23%, and 42%, respectively. Eighty-three percent had only pelvic, 2% had only paraaortic, and 15% had pelvic and paraaortic LN metastases. Cumulatively, a total dose converted to EQD2_1.5 Gy_ of 66 Gy (60–70 Gy) was delivered to the prostatic fossa, 70 Gy (66–72 Gy) to the local recurrence, if present, 65.1 Gy (56–66 Gy) to PET-positive lymph nodes, and 47.5 Gy (42.4–50.9 Gy) to the lymphatic pathways. Concomitant androgen deprivation therapy (ADT) was administered in 83% of patients. One-, 2-, and 3-year BRFS was 80.7%, 71.6%, and 65.8%, respectively. One-, 2-, and 3-year DMFS was 91.6%, 79.1%, and 66.4%, respectively. In multivariate analysis, concomitant ADT, longer ADT duration (≥ 12 vs. < 12 months) and LN localization (pelvic vs. paraaortic) were associated with improved BRFS and concomitant ADT and lower PSA value before sRT (< 1 vs. > 1 ng/ml) with improved DMFS, respectively. No such association was seen for the number of affected lymph nodes.

**Conclusions:**

Overall, the present analysis shows that the so far, unmatched sensitivity and specificity of PSMA-PET/CT translates in comparably high BRFS and DMFS after PSMA-PET/CT-based sENRT for patients with PCa LN recurrence. Concomitant ADT, duration of ADT, PSA value before sRT, and localization of LN metastases were significant factors for improved outcome.

## Background

About one-third of patients treated with radical prostatectomy (RP) for prostate cancer (PCa) experience biochemical persistence (bcP) or biochemical recurrence (bcR) [1, 2]. In this situation, postoperative radiotherapy (RT) is a well-established treatment option even though there are still some ongoing questions. Surveys among radiation oncologists show a wide variety of treatment protocols [3, 4]. In particular, timing of RT, dose prescription, treatment volume, use of concomitant androgen deprivation therapy (ADT), and the choice of pre-treatment imaging are essential topics still under discussion. Regarding the last point, prostate-specific membrane antigen positron emission tomography/computed tomography (PSMA-PET/CT) prevailed against choline PET/CT and fluciclovine PET/CT. It is therefore viewed as the gold standard for staging in this situation and is recommended by EAU guidelines [5]. PSMA-PET/CT is known to have a high positive predictive value [6] and to detect PSMA-positive lesions already at low prostate-specific antigen (PSA) levels thus leading to a high amount of treatment modifications [7–9]. Furthermore, studies evaluating PSMA-PET/CT staging in different clinical situations showed a high efficacy in patients with bcR and bcP with a PSMA positivity rate of more than 40% and more than 60%, respectively [10, 11]. Overall, positive results in PSMA-PET/CT staging are a good predictor for failure-free survival in patients with a biochemical relapse after RP [12]. Often, PSMA-PET/CT imaging reveals lymph node (LN) recurrence in patients with bcP or bcR [13–15]. Also in this subset of patients, RT represents one of the mainstays of treatment even though the radiotherapeutic approach (stereotactic body radiotherapy (SBRT) vs. elective nodal radiotherapy (ENRT)), the role of concomitant ADT and the therapeutic consequences of paraaortic LN involvement are still to be evaluated. As shown in mostly retrospective analyses so far, ENRT seems to be an effective treatment strategy with rare side effects [16–19]. Most of these analyses included mostly patients prior to the PSMA-PET/CT era and so data on PSMA-PET/CT-based salvage ENRT (sENRT) for LN recurrence after RP are currently still sparse. Thus, this retrospective study reports on the outcome after PSMA-PET/CT-based sENRT in patients with pelvic and/or paraaortic LN recurrence. Furthermore, this analysis aims to investigate the benefit of additional ADT in patients with LN recurrence in order to facilitate patients’ counseling.

## Methods

### Patient population

Patients consecutively undergoing PSMA-PET/CT-based sENRT for LN recurrence at the University Hospital, LMU Munich, were considered for this analysis and were retrospectively analyzed. All patients had histologically confirmed PCa and were referred for sRT after RP due to persistent or rising PSA. No patient had prior RT to the prostate or the prostate bed. All patients provided written informed consent to undergo PSMA PET/CT. An interdisciplinary tumor board approved the treatment indication. Patients with M1a disease limited to the lumboaortic region below the renal arteries were included, while patients with M1b/M1c disease were excluded. This retrospective analysis was performed in compliance with the principles of the Declaration of Helsinki and its subsequent amendments [20] and was approved by the local Ethics Committee of the Medical Faculty (approval number 19–361).

### PSMA ligand and PET/CT imaging protocol

Pretreatment imaging was performed with ^68^ Ga- or ^18^F-labeled PSMA-PET/CT in 75% (^68^ Ga-PSMA-11) and 25% (^18^F-PSMA-1007) of patients, respectively. Radiolabeling was performed according to good clinical practice as described previously [21, 22]. A Siemens Biograph 64 or GE Discovery 690 PET/CT scanner was used for PSMA-PET/CT imaging. Phantom studies based on the National Electrical Manufacturers Association NU2-2001 standard were conducted in Munich to allow for pooling of the different scanner results. At the time of the PET scan, a contrast-enhanced diagnostic CT (120 kV, 100–400 mAs, dose modulation) or a low-dose CT (120 kV, 25 mAs) for attenuation correction (depending on previous CT scans and contraindications) was performed. PSMA-PET/CT scans were acquired approx. 60 min after intravenous injection of the ^68^ Ga-/^18^F-PSMA-ligand complex. Barring any contraindications, patients receiving PSMA-PET/CT were administered 20 mg furosemide at the time of tracer injection to avoid bladder activity and to reduce radiation exposure.

### Image analysis

PET/CT was interpreted by one nuclear medicine physician and one radiologist in the sense of a clinical report-based analysis. Both readers had more than 5 years of PET/CT experience. Location of lesions was each determined by CT. PET-positive lesions were visually identified by ^68^ Ga-/^18^F-PSMA uptake above background and not associated with the physiologic uptake [23].

### Radiotherapy treatment and follow-up

All patients were treated with intensity modulated RT (IMRT) or volumetric arc therapy (VMAT) with 5 fractions per week. Image-guided RT (IGRT) was performed with cone-beam CT (2–5 times per week). RT dose regimens were normo- or slightly hypofractionated with a simultaneous integrated boost (SIB) to the PET-positive lesions. Target delineation was performed according to the Radiation Therapy Oncology Group (RTOG) atlas for salvage PCa and for pelvic LN delineation and was extended in case PSMA-PET/CT revealed pathological LN outside the recommended clinical target volume [24]. ADT was recommended to all patients for 24–36 months, but the duration could be adjusted at the discretion of the treating urologist depending on comorbidities, side effects, and patient’s preference. Follow-up examination was first carried out 3 months after RT and then every six to 12 months.

### Endpoints and statistical analysis

The primary endpoint was biochemical recurrence-free survival (BRFS) defined as PSA < post-RT nadir + 0.2 ng/ml. The secondary endpoint was distant metastasis-free survival (DMFS). Survival data were calculated as time from last day of RT to biochemical progression/diagnosis of distant metastasis or to the date of the last follow-up. Statistical analyses were conducted using IBM-SPSS® version 26.0. Survival analyses were calculated using the Kaplan–Meier method and compared using the log-rank test. For multivariate analysis, cox regression analysis was performed including potential covariates such as ISUP score, initial tumor stage, initial nodal stage, number of PSMA PET-positive LN metastases, PSMA PET-positive LN localization, presence of PSMA PET-positive local recurrence, PSA before sRT, concomitant ADT, duration of ADT, and PSA persistence vs. PSA recurrence. A *p*-value < 0.05 was considered significant.

## Results

Between April 2014 and December 2019, 100 patients with bcP (76%) or bcR (24%) after RP underwent PSMA-PET/CT-based RT for LN recurrence. Median follow-up was 37.6 months. Patient characteristics are shown in Table 1. Initial tumor stage was pT2 in 21% and ≥ pT3 in 79%. Initial nodal stage was pN0 in most patients (54%). Fifty-two percent had positive surgical margins after RP. Most patients had International Society of Urological Pathology (ISUP) score 4 or 5 (61%). The median PSA-Nadir after RP was 0.6 ng/ml.

**Table 1:** Patient characteristics

Patients, *n*	100
Age, median (range)	72 (46–82)
Initial tumor stage, *n* (%)	
pT2a	1 (1%)
pT2c	20 (20%)
pT3a	25 (25%)
pT3b	51 (51%)
pT4	3 (3%)
Initial nodal stage, *n* (%)	
pN0	54 (54%)
pN1	42 (42%)
pNx/cN0	2 (2%)
Unknown	2 (2%)
Positive surgical margins, *n* (%)	52 (52%)
ISUP score, *n* (%)	
1	2 (2%)
2	12 (12%)
3	25 (25%)
4	18 (18%)
5	43 (43%)
PSA at RP (ng/ml), median (range)	13.9 (0.05–427)
Postoperative PSA (ng/ml), median (range)	0.6 (0.0–40.1)
Patients with bcP/bcR, *n* (%)	76 (76%)/24 (24%)
Time between RP and bcR (month), median (range)	22.5 (2–148)
PSA at PSMA-PET/CT (ng/ml), median (range)	1.4 (0.1–40.1)
PSA at PSMA-PET/CT in patients with bcP (ng/ml), median (range)	1.7 (0.1–40.1)
PSA at PSMA-PET/CT in patients with bcR (ng/ml), median (range)	0.6 (0.3–5.1)
Number of lymph node metastases on PSMA-PET/CT, *n* (%)	
1	35 (35%)
2	23 (23%)
≥ 3	42 (42%)
Patients with local recurrence on PSMA-PET/CT, *n* (%)	29 (29%)

*Abbreviations*:* bcP* biochemical persistence; *bcR* biochemical recurrence; *ISUP* International Society of Urological Pathology; *n* number; *PSA* prostate-specific antigen; *RP* radical prostatectomy

The median interval between surgery and bcR was 22.5 months. The median PSA at the time of the PSMA-PET/CT was 1.7 ng/ml (range 0.1–40.1 ng/ml) in patients with bcP and 0.6 ng/ml (range 0.3–5.1 ng/ml) in patients with bcR. PSMA-PET/CT showed one, two, and three or more LN metastases in 35%, 23%, and 42%, respectively. Eighty-three percent had pelvic only, 2% had paraaortic only, and 15% had pelvic and paraaortic LN metastases.

Treatment characteristics are shown in Table 2. Median RT doses converted to EQD2 equivalent doses using an α/β ratio of 1.5 Gy were 47.5 Gy (range 42.4–50.9 Gy) to the pelvic lymphatic drainage pathways, 66 Gy (range 60–70 Gy) to the prostatic fossa, and if present, 70 Gy (66–72 Gy) to PSMA PET-positive local recurrence. PET-positive lymph nodes received a SIB of median 65.1 Gy (range 56–66 Gy). ADT was recommended to all patients but refused by 17%, resulting in concomitant administration in 83% of patients. The duration of the ADT was less than 6 months, 6 to 12 months, 12 to 24 months, and more than 24 months in 18%, 22%, 17%, and 22% of the patients, respectively. Eighty percent of patients had no ADT at last follow-up and time between end of ADT and last follow-up was in median 31.9 months.

**Table 2:** Treatment characteristics

ADT	
Concomitant ADT	83 (83%)
Duration ADT	
< 6 months	18 (18%)
6–12 month	22 (22%)
12–24 month	17 (17%)
> 24 months	22 (22%)
Unknown	4 (4%)
RT	
Dose to prostatic fossa EQD2_1.5 Gy_ (Gy), median (range)	66 (60–70)
Dose to local recurrence EQD2_1.5 Gy_ (Gy), median (range)	70 (66–72)
Dose to lymphatic pathways EQD2_1.5 Gy_ (Gy), median (range)	47.5 (42.4–50.9)
Dose to PET-pos. lymph nodes EQD2_1.5 Gy_ (Gy), median (range)	65.1 (56–66)

*Abbreviations*:* ADT* androgen deprivation therapy; *RT* radiotherapy

**Fig. 1:**
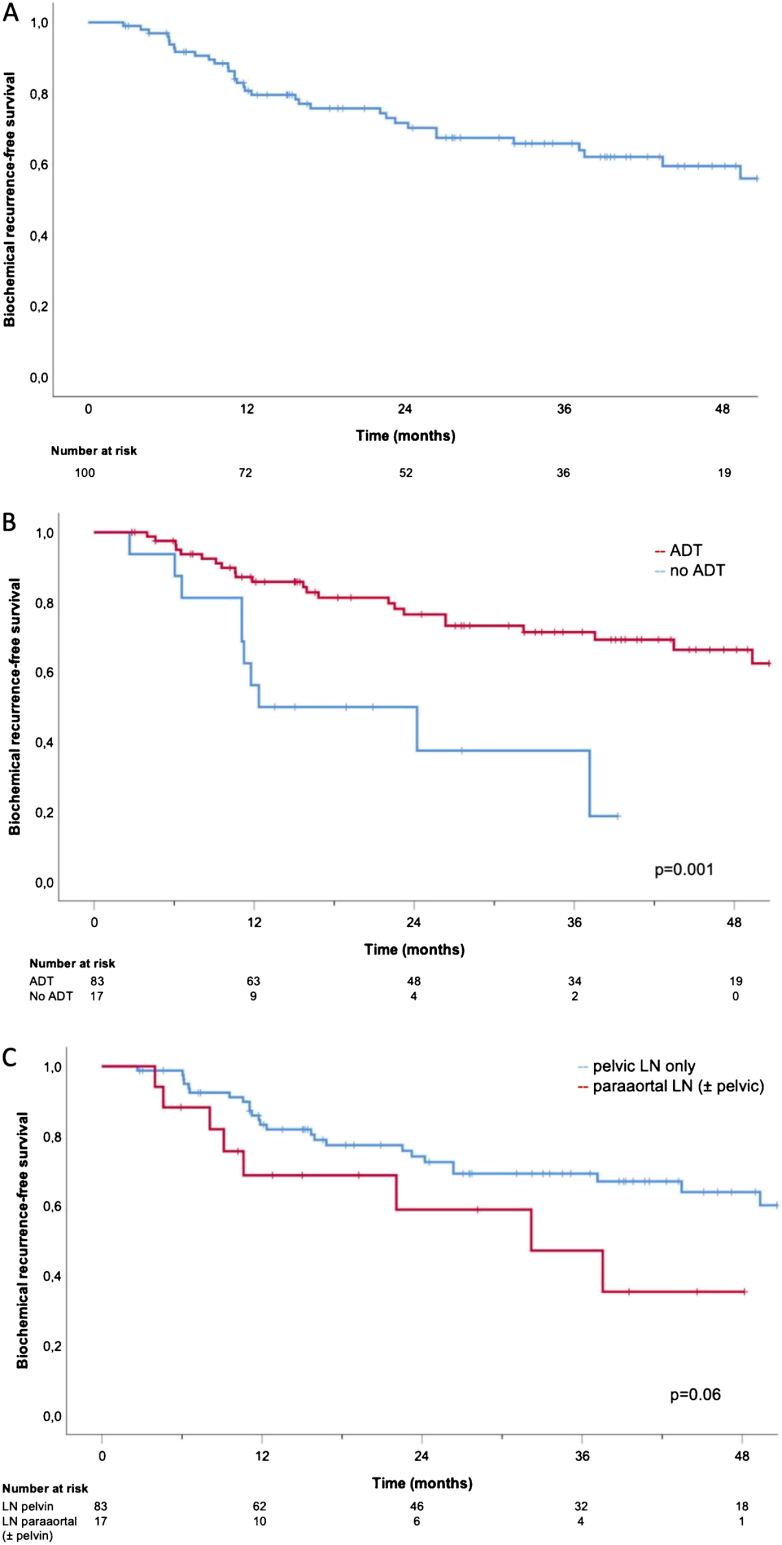
Kaplan–Meier curves: BRFS (**A**), BRFS for ADT vs. no ADT (**B**), and BRFS for pelvic lymph nodes vs. paraaortic (± pelvic) lymph nodes (**C**)

**Fig. 2:**
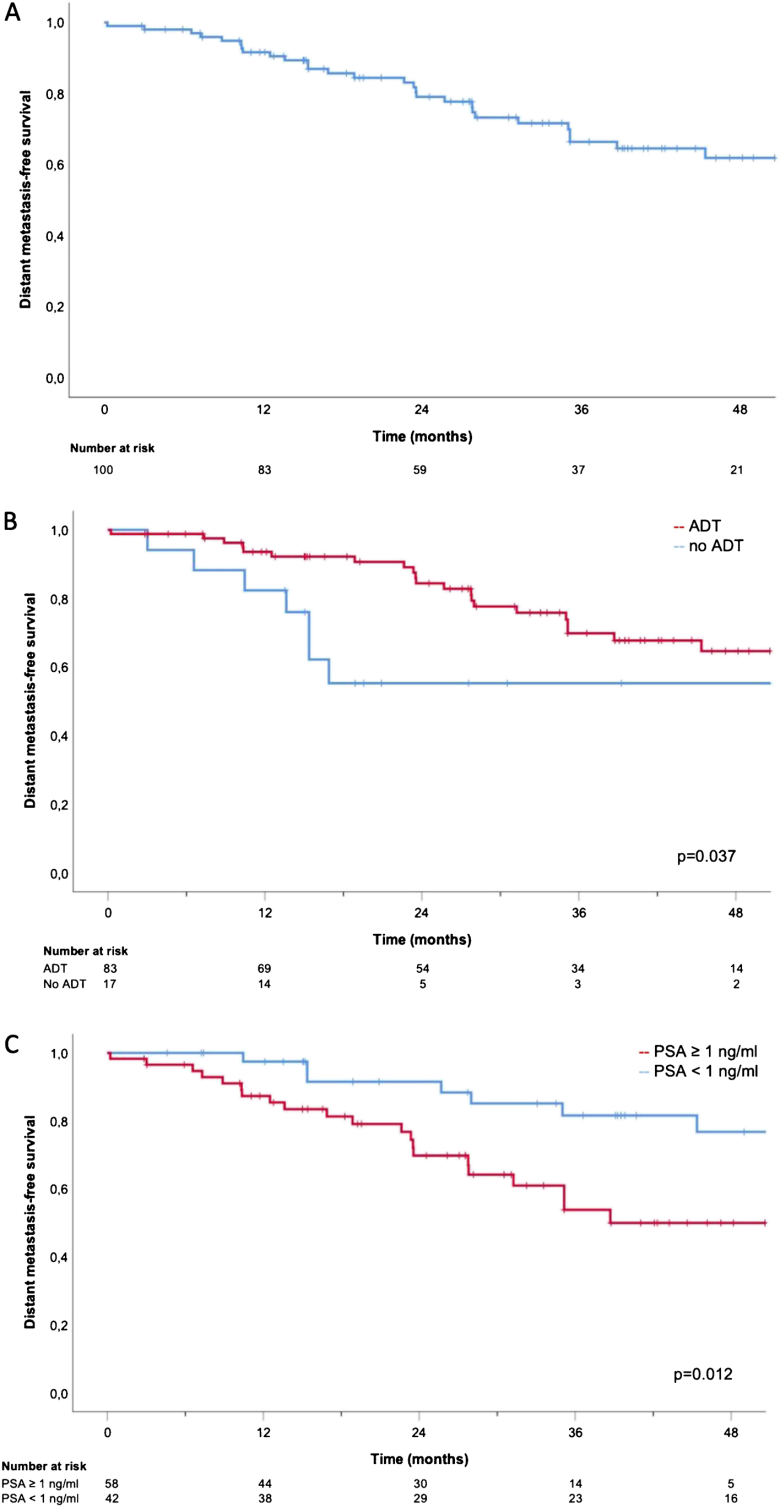
Kaplan–Meier curves: DMFS (**A**), DMFS for ADT vs. no ADT (**B**), and DMFS for PSA pre-sRT < 1 vs. ≥ 1 ng/ml (**C**)

Median BRFS was not reached. One-, 2-, and 3-year BRFS was 80.7%, 71.6%, and 65.8%, respectively (Fig. 1). In those patients without ADT at last follow-up (80/100), 1-, 2-, and 3-year BRFS was 77.6%, 67.6%, and 61.5%, respectively. Median DMFS was not reached. One-, 2-, and 3-year DMFS was 91.6%, 79.1%, and 66.4%, respectively (Fig. 2). In patients without ADT at last follow-up, 1-, 2-, and 3-year DMFS was 90.9%, 76.9%, and 63.6%. Tables 3 and 4 show the results of the univariate and multivariate analyses. Concomitant ADT was significantly associated with improved BRFS and DMFS whereas the duration of ADT treatment (< 12 vs. ≥ 12 months) was only significantly associated with BRFS but not with DMFS. A PSA value < 1 ng/ml before sRT was significantly associated with better BRFS (only in univariate but not in multivariate analysis) and DMFS. Paraaortic LN localization was associated with worse BRFS in multivariate analysis.

**Table 3:** Uni- and multivariate analysis for factors associated with BRFS

Patient characteristics	*n*	Univariate		Multivariate	
		Median BRFS	p-value	HR (95% CI)	*p*-value
ISUP score			0.580	1.09 (0.45–2.63)	0.854
≤ 3	39	NR			
≥ 4	61	NR			
Initial tumor stage			0.367	2.06 (0.51–8.24)	0.308
≤ T2	21	NR			
≥ T3	79	NR			
Initial nodal stage			0.538	0.90 (0.40–2.03)	0.798
N0	54	NR			
N1	42	NR			
Number of lymph node metastases			0.277	1.47 (0.87–2.49)	0.147
1	35	NR			
2	23	NR			
≥ 3	42	NR			
Lymph node localization			0.060	2.97 (1.10–8.04)	**0.032**
Pelvic	83	NR			
Paraaortic (± pelvic)	17	32.2			
Concomitant ADT			**0.001**	0.26 (0.09–0.76)	**0.013**
present	83	NR			
Absent	17	12.4			
ADT duration			**0.021**	0.28 (0.11–0.73)	**0.009**
≤ 12 months	40	37.2			
> 12 months	39	NR			
PSA persistence vs. PSA recurrence			0.17	1.78 (0.47–6.74)	0.396
PSA persistence	76	NR			
PSA recurrence	22	NR			
PSA before sRT			**0.027**	1.92 (0.81–4.57)	0.140
< 1 ng/ml	42	NR			
≥ 1 ng/ml	58	NR			
Local recurrence			0.336	1.14 (0.48–2.72)	0.767
No local recurrence	71	NR			
Local recurrence	29	37.6			

*Abbreviations*:* ADT* androgen deprivation therapy; *CI* confidence interval; *HR* hazard ratio; *ISUP* International Society of Urological Pathology; *NR* not reached; *PSA* prostate-specific antigen; *sRT* salvage radiotherapy

Bold values denote statistical significance at the *p* < 0.05 level

**Table 4:** Uni- and multivariate analysis for factors associated with DMFS

Patient characteristics	*n*	Univariate		Multivariate	
		Median DMFS	*p*-value	HR (95% CI)	*p*-value
ISUP score			0.929	1.45 (0.56–3.71)	0.442
≤ 3	39	NR			
≥ 4	61	NR			
Initial tumor stage			0.456	1.02 (0.26–4.08)	0.978
≤ T2	21	NR			
≥ T3	79	NR			
Initial nodal stage			0.685	0.75 (0.32–1.73)	0.491
N0	54	NR			
N1	42	NR			
Number of lymph node metastases			0.447	1.21 (0.72–2.02)	0.469
1	35	NR			
2	23	54.8			
≥ 3	42	53.9			
Lymph node localization			0.276	1.39 (0.49–3.93)	0.534
Pelvic	83	NR			
Paraaortic (± pelvic)	17	32.2			
Concomitant ADT			**0.037**	0.25 (0.07–0.89)	**0.033**
Present	83	NR			
Absent	17	12.4			
ADT duration			0.342	0.84 (0.35–2.03)	0.693
≤ 12 months	40	NR			
> 12 months	39	NR			
PSA persistence vs. PSA recurrence			0.152	2.26 (0.51–9.90)	0.281
PSA persistence	76	61.6			
PSA recurrence	22	NR			
PSA before sRT			**0.012**	2.56 (1.01–6.46)	**0.047**
< 1 ng/ml	42	NR			
≥ 1 ng/ml	58	38.7			
Local recurrence			0.285	0.90 (0.37–2.19)	0.814
No local recurrence	71	NR			
Local recurrence	29	45.4			

*Abbreviations*:* ADT* androgen deprivation therapy; *CI* confidence interval; *HR* hazard ratio; *ISUP* International Society of Urological Pathology; *NR* not reached; *PSA* prostate-specific antigen; sRT salvage radiotherapy

Bold values denote statistical significance at the *p* < 0.05 level

## Discussion

The present analysis shows a high 2- and 3-year BRFS of 72% and 66% and a high 2- and 3-year DMFS of 79% and 66% after PSMA-PET/CT-based sENRT for such an oncologically unfavorable group of patients with PCa LN recurrence with thus a potentially second chance of cure [25]. To account for the bias of an ongoing ADT on these endpoints, we assessed survival data separately for 80/100 patients without ongoing ADT at last follow-up. In these patients, 2- and 3-year BRFS was 68% and 62%, and 2- and 3-year DMFS was 77% and 64%, respectively. Multivariate analysis reconfirmed the use of concomitant ADT, the duration of ADT, the PSA level before sRT and the LN localization as significant factors for an improved outcome.

Overall, these results are very promising in comparison to other studies reporting on PET/CT based ENRT with SIB to macroscopic LN metastases. In 2017, Fodor et al. published a retrospective analysis of 81 patients with LN recurrence and reported a 3-year BRFS of 42% and a 3-year clinical relapse-free survival of 62%. The main differences to the current analysis are a visibly more heterogenous cohort with more than half of the patients with previous RT and even inclusion of patients with mediastinal lymph nodes as well as the use of choline PET/CT as staging prior to RT [16]. In 2018, Tran et al. retrospectively analyzed 53 patients with pelvic or paraaortic LN recurrence. They described a 5-year BRFS of 42%. In contrast to the present analysis, all patients received ADT and RT was based on nowadays fairly outdated ^11^C-acetate or ^18^F-choline PET/CT [17]. Recently, Ingrosso et al. reported on 41 patients with pelvic LN metastases with a 3-year BRFS of 53% and a 3-year radiological progression-free survival of 64%. Again, their study differed from the present analysis in regard to a more heterogenous cohort of patients (almost 40% with RT beforehand), the primarily use of choline PET/CT as staging method, and another definition of PSA relapse (rise to more than 25% above the PSA value pre-sRT) [18].

The prospective “Oligopelvis-GETUG-P07” phase II trial reported a 2- and 3-year progression-free survival (PFS) of 81% and 51%, respectively, and a 2- and 3-year BRFS of 58% and 46%, respectively, in 67 patients with pelvic LN relapse. Consistently with the so far mentioned studies, staging prior to RT was based on choline PET/CT. All patients received 6 months ADT and half of the patients had prior prostate or prostate bed RT. Progression was again defined differently as two consecutive PSA levels above the level at inclusion or clinical progression thus hampering a direct comparison [19]. In contrast to the mentioned studies evaluating mainly patients with choline PET/CT-based sENRT [16–19], patients in the present analysis received PSMA-PET/CT-based sENRT which probably explains the comparably good outcomes in this analysis.

Nevertheless, despite the promising results of these analyses, the best therapeutic strategy in patients with LN recurrence is still a matter of debate [26] and data comparing ENRT with other treatment strategies are so far sparse.

A possible treatment strategy could be ADT alone with data from the prospective “Oligopelvis 2-GETUG P12” phase III trial, randomizing patients with LN oligorecurrence between ADT alone and ADT plus PET/CT-based ENRT, still pending and eagerly awaited. Another possible treatment strategy is SBRT. There is growing evidence for SBRT in patients with LN metastases coming from trials which evaluated SBRT as metastasis-directed therapy (MDT) in oligometastatic PCa and which included patients with nodal oligometastases [27–30]. Moreover, there is a retrospective analysis of SBRT in patients with nodal oligorecurrence [31]. In all these studies, the stereotactic approach showed good local control and low toxicity rates. Nevertheless, compared to ENRT, PFS in these studies seems to be worse with a 2-year PFS between 16 and 58% mainly triggered by LN recurrence outside the radiation volume. This has been reconfirmed in a large retrospective analysis with more than 500 patients by De Bleser et al. directly comparing SBRT to ENRT in patients with LN recurrence. They analyzed more than 500 patients with nodal oligorecurrence staged mostly by choline PET/CT and treated by either ENRT (with or without SIB) or SBRT. Patients had a significantly worse 3-year metastasis-free survival of 68% when treated with SBRT compared to 77% after ENRT [32]. However, a considerable number of patients in the ENRT cohort already had prior ADT (32%) or prior RT of the prostate, the prostate bed, or the whole pelvis (66%) which complicates a direct comparison with our data and might explain the better results. The benefit of a node-based therapeutic approach is currently being analyzed in the prospective randomized “STORM” phase II trial. Patients with pelvic LN oligorecurrence receive MDT with salvage lymph node dissection (sLND) or SBRT plus 6 months of ADT with or without additional ENRT. The study is expected to be completed at the end of 2023 and will further clarify this issue [33]. Furthermore, retrospective data suggest a trend towards a better disease-free survival and a significantly lower ADT administration rate for PSMA-PET/CT-based SBRT compared to choline PET/CT-based SBRT [34].

So far, there is limited evidence that adjuvant ENRT also improves the relapse-free survival in patients treated with sLND compared to sLND alone when staged with choline PET/CT [35]. When comparing in retrospective studies sLND with ENRT in patients with LN recurrence all staged with PSMA-PET/CT, there was an equally higher BRFS in the ENRT cohort [36, 37]. Therefore, the hitherto mainly short-term oncological data of patients with LN recurrence in the current PSMA-PET/CT era indicate a benefit for a more extensive therapeutic approach as it is the case with ENRT.

In the present PSMA-PET/CT-based ENRT, cohort concomitant use of ADT was a strong predictor not only for better BRFS in uni- and multivariate analysis, but also for improved DMFS. In other studies evaluating PET/CT-based ENRT in LN recurrence, there was either no effect of concomitant ADT [16, 18] or all patients received ADT [17, 19]. High-quality prospective data is until now only available for patients with bcR, in whom the real number of patients with possibly LN recurrence is mainly unknown. Overall, the addition of ADT significantly improved not only BRFS, but also DMFS and even overall survival (OS) in these studies [38–40]. Regarding the duration of ADT, in the present analysis, there was a significant association with better BRFS but not with DMFS. So far, there is no high-quality evidence from prospective studies addressing the question of ADT duration in the setting of postoperative RT. The LOBSTER trial randomizing between 6 and 24 months of concomitant ADT in patients with bcR receiving sRT of the prostate will shed light on this question [41]. Further research is needed to clarify the ideal duration of ADT in the subset of patients with LN metastases and sENRT. Even in that subset of patients, it might make sense to distinguish between patients with different risk profiles, analogous to duration of concomitant ADT in the definitive prostate RT setting. Apart from concomitant ADT, a PSA < 1 ng/ml prior to sRT correlated with an improved DMFS in uni- and multivariate analysis as well as with an improved BRFS in the univariate analysis. Lower PSA prior to sRT is a well-known factor associated with better BRFS as proven in retrospective studies [42] and as recently shown in three prospective studies on early sRT [43–45]. In other studies explicitly evaluating ENRT in patients with LN recurrence, the predictive power of the PSA value was either not reported [16, 17, 46] or did not reach significance in the multivariate analysis [18]. However, a PSA doubling time of less than 3 months at the time of recurrence seems to have a negative impact on the outcome [17].

Regarding BRFS, also the LN localization was a significant predictor in multivariate analysis: paraaortic LN involvement was, as one might expect, associated with a worse outcome. Fodor et al. also observed a significant association between extrapelvic LN metastases and higher clinical relapse rates in patients treated with PET/CT-based sENRT [16]. The observed prognostic relevance reflects in the TNM classification which classifies paraaortic LN involvement already as distant metastases (M1a) [47]. In the present cohort, longer follow-up is probably needed to gain insight into the influence of paraaortic LN metastases on DMFS.

Apart from those mentioned, there were no other significant predictors for BRFS in our analysis. In particular, there was no difference between patients with bcP and bcR. Contrary to the present findings, RT indication (bcP vs. bcR) was observed as a viable predictor for a PSA ≤ 0.2 ng/ml at last follow-up in patients receiving PSMA-PET-guided sRT in a retrospective analysis [22]. Maybe this effect is masked in patients with LN metastases due to their overall worse prognosis. Longer follow-up and more balanced groups will be necessary to further clarify this question also in the present subset of patients.

To our best knowledge, this study is so far the largest analysis reporting on the outcome of PSMA-PET/CT-based sRT using ENRT with SIB to LN metastases in patients with nodal recurrence. A comparatively homogenous patient cohort was analyzed after exclusion of patients with PET-positive bone metastases or previous RT of the prostate or prostatic fossa.

However, the current study is not without limitations mostly due to its retrospective character: Especially, the use and duration of ADT differed between patients in the present analysis mirroring patients’ preferences in a real-word setting. Furthermore, a median follow-up time of more than 3 years is until now not long enough to report on other endpoints, such as cancer-specific survival and OS. Moreover, treatment-related toxicity which needs to be taken into account when choosing a treatment strategy is not reported in this analysis.

## Conclusions

Overall, the present analysis shows that the high rate of treatment modifications due to the so far unmatched sensitivity and specificity of PSMA PET/CT translates in a comparably high 2- and 3-year BRFS of 72% and 66% and 2- and 3-year DMFS of 79% and 66% after PSMA-PET/CT-based sENRT for patients with PCa LN recurrence. Significant factors for an improved outcome were use of concomitant ADT, duration of concomitant ADT > 12 months, PSA before sRT < 1 ng/ml, and the absence of paraaortic LN metastases. As median BRFS and DMFS are still not reached, longer follow-up and above all randomized controlled data is needed to further implement sENRT as treatment in patients with PET-positive LN recurrence.

## Data Availability

Research data are stored in an institutional repository and will be shared upon request to the corresponding author.

## Abbreviations

ADT: Androgen deprivation therapy
bcP: Biochemical persistence
bcR: Biochemical recurrence
BRFS: Biochemical recurrence-free survival
CT: Computed tomography
CI: Confidence interval
DMFS: Distant metastasis-free survival
ENRT: Elective nodal radiotherapy
HR: Hazard ratio
IMRT: Intensity modulated radiotherapy
ISUP: International Society of Urological Pathology
IGRT: Image-guided radiotherapy
LN: Lymph node
MDT: Metastasis directed therapy
NR: Not reached
*n*: Number
OS: Overall survival
PFS: Progression free survival
PCa: Prostate cancer
PSA: Prostate-specific antigen
PSMA-PET/CT: Prostate-specific membrane antigen positron emission tomography/computed tomography
RP: Radical prostatectomy
RTOG: Radiation therapy oncology group
RT: Radiotherapy
sENRT: Salvage elective nodal radiotherapy
sLND: Salvage lymph node dissection
sRT: Salvage radiotherapy
SIB: Simultaneous integrated boost
SBRT: Stereotactic body radiotherapy
VMAT: Volumetric arc therapy
